# Coping strategies in a sample of anxiety patients: factorial analysis and associations with psychopathology

**DOI:** 10.1002/brb3.351

**Published:** 2015-06-24

**Authors:** Gino Pozzi, Alessandra Frustaci, Daniela Tedeschi, Silvia Solaroli, Paolo Grandinetti, Marco Di Nicola, Luigi Janiri

**Affiliations:** 1 Institute of Psychiatry and Psychology, Catholic University of Sacred HeartLargo F. Vito 1, 00168, Rome, Italy; 2 Unit of Clinical and Molecular Epidemiology, IRCCS San Raffaele PisanaVia di Val Cannuta 247, 00166, Rome, Italy; 3 L.U.M.S.A., Libera Università Maria SS. AssuntaPiazza delle Vaschette 101, 00193, Rome, Italy

**Keywords:** Anxiety disorders, Brief-COPE inventory, coping, cross-sectional study

## Abstract

**Background:**

The relationship between coping styles and mental disorders has received considerable attention and instruments have been developed to assess coping strategies. The measurement by means of category systems has been criticized and a functional hierarchy of action types linked to the adaptive processes is preferred. We aimed to determine which factors may exist within the Brief-COPE (Brief Coping Orientation to Problems Experienced – COPE – Inventory) in an Italian sample of patients with anxiety disorders; and if these factors correlate with the severity of psychopathology or with other characteristics.

**Methods:**

A total sample of 148 patients was recruited. The Brief-COPE inventory, the Symptom Check List 90-Revised, the Penn State Worry Questionnaire, the Zung Anxiety Status Inventory and the Zung Self-Rating Anxiety Scale were administered.

**Results:**

Factor analysis of the Brief-COPE yielded nine factors accounting for 65.48% of the variance. Patients scored higher on Searching Support, followed by Acceptance, Changing Perspective, and Problem Solving. Associations between measures of psychopathology and factors of coping strategies, mostly Searching support and Avoidance, were found.

**Conclusions:**

Data of the present study support a nine-factor structure of the Brief-COPE that includes five broad dimensions of coping. Psychopathology was mostly related to Searching support and Avoidance factors, showing that these strategies may reflect ineffective ways of coping; Problem solving and Changing perspective could be a valid approach to moderate anxiety/depression symptoms and psychopathology in general.

## Introduction

In origin, coping was seen as an action directed to the resolution or mitigation of a problematic situation, as seen in animal experimentation (Ray et al. 1982; Lazarus and Folkman 1991). In a stress model, coping is viewed as a major component of the overall stress process, and is treated as a mediating link between stressors and psychological strain, or as a moderator of the stress–strain relationship (Ogden 2000).

Although a universally accepted understanding of the many possible ways by which people cope is not recognized, coping styles are classified according to the prevailing strategies used to face stressful life events, so that different characterization of coping strategies emerge, namely “dichotomous models”, that is, problem-focused versus emotion-focused coping (Ogden 2000), approach versus avoidance coping, or a “three-categorical classification” of behavioral, cognitive, and emotional coping domains (Schwarzer and Schwarzer 1996).

There is a general consensus from empirical studies that task-oriented (or “problem-focused”) coping in response to stressful life events is associated with lower levels of emotional distress than other coping styles. Several studies report that the effective use of problem-focused coping strategies may lower distress even in patients with advanced somatic disease (Uitterhoeve et al. 2004; Naaman et al. 2009).

The relationship between coping styles and anxiety, has received considerable attention in a number of studies since 80s (Folkman and Lazarus 1986). Concerning Anxiety Disorders (ADs), the predominance of dysfunctional coping styles in these patients has been reported (Vollrath and Angst 1993; Katerndahl 1999; Hino et al. 2002; Thomasson and Psouni 2010; Legerstee et al. 2011). Coping styles may contribute to anxiety vulnerability (Ouimet et al. 2009), and may be strong predictors of anxiety symptomatology, with greater emotion-oriented coping in patients with high levels of anxiety (Uehara et al. 2002; Kraaij et al. 2003; Matheson and Anisman 2003). Avoidance coping has been reported as the prevailing style in AD patients. More specifically, in phobic patients avoidance coping is prevailing as compared to nonclinical samples (Davey et al. 1995). Similarly, among panic sufferers, various studies reported that avoidant coping is the most frequently used coping style (Vitaliano et al. 1087; Hughes et al. 1999), a premorbid predictor of anxious responding to panic-like bodily sensations (Feldner et al. 2004), the self-perceived most effective way to deal with anxiety-related concerns (Cox et al. 1992) and a risk factor for the development of panic symptoms (Kaplan et al. 2012).

The measurement of coping by means of category systems has been reviewed and criticized, leading to the conclusion that dichotomous distinctions (i.e., problem- vs. emotion-focused, approach vs. avoidance, cognitive vs. behavioral) should be replaced by a functional hierarchy of action types linked to the adaptive processes at any level (cognitive, emotional, behavioral, relational) (Endler and Parker 1990; Skinner et al. 2003). One of the instrument developed which is used widely is the Coping Orientation to Problems Experienced (COPE) inventory (Carver et al. 1989, 1993; Sica et al. 1997a,b). COPE is primarily a theoretically derived measure (such as proposed coping strategies are not derived empirically and then linked post hoc by factorial analysis, but rationally), based on Carver and Scheier’s self-regulation theory (Carver and Scheier 1981, 1982), it makes several distinctions within the overall categories of problem-focused and emotional-focused coping (Sica et al. 1997a) and holds that individuals make decisions and act upon them in ways that reduce the gap between actual and desired outcomes, or goals. Although the COPE measures were designed to assess more fine-grained aspects of coping, factor analytic studies have demonstrated that broader dimensions of coping also exist (Kapsou et al. 2010). Furthermore, failures to complete the whole measure, observed frustration of participants, and other problems in administration (Carver et al. 1993; Carver 1997) led to the development of a less extensive version, the Brief-COPE (Carver 1997), which is increasingly used in research (Paukert et al. 2009; Kapsou et al. 2010; Bautista and Erwin 2013; Bautista et al. 2013). With the exception of a couple of scales, the instrument presents good reliability (Carver 1997; Muller and Spitz 2003; Kapsou et al. 2010) and was translated in several languages, including Italian (Conti 2000; Perna et al. 2007), Greek, (Kapsou et al. 2010), Spanish (Perczek et al. 2000), and French (Muller and Spitz 2003). The Brief-COPE is not designed to obtain an overall score and its Author suggests that users can create factors using their own data (Carver 2007).

The purpose of our study was to determine what factors exist within the Brief-COPE inventory in an Italian sample of patients with ADs, and, as we hypothesize that ADs patients may use dysfunctional coping styles, if these factors are associated with the severity of psychopathology.

## Materials and Methods

### Sampling

Patients suffering from ADs were consecutively recruited from a tertiary-level outpatient clinic at the University General Hospital “A. Gemelli” in Rome from 2008 to 2012. Inclusion criteria were: (1) fulfilling diagnostic criteria of any DSM-IV-TR anxiety disorder (American Psychiatric Association, 2000); (2) age between 18 and 65 years; (3) having at least primary school education; (4) the property of written and spoken Italian language. Exclusion criteria were: (1) an inability or unwillingness to cooperate; (2) a diagnosis of mental retardation or documented IQ < 70; (3) history positive for serious medical diseases; (4) mental disorders due to substance-related or medical condition; (5) having any other current or past Axis I or II mental disorders.

Patients’ interviews were part of the routine assessment at intake; Axis I diagnosis was preliminary established by trained psychiatrists using the Structured Clinical Interview for DSM-IV Axis I Disorders (SCID-I) (First et al. 1995); also, over-threshold personality disorders were ruled out according to DSM-IV-TR criteria (American Psychiatric Association, 2000).

After the establishment of the AD diagnosis, an anamnestic interview was administered to obtain sociodemographic information, medical and psychiatric history, and familiar history of psychopathology. At the evaluation session, patients were drug naïve, drug free, or undergoing an inadequate pharmacological treatment given that residual symptoms persisted.

The study protocol was conducted in accordance with Good Clinical Practice guidelines and the Declaration of Helsinki (1964) and subsequent revisions. Subjects gave informed consent to be recruited in the study, for which they voluntarily participated without receiving any form of payment. Anonymity was guaranteed to all the participants for research purposes.

### Instruments

Each patient was administered a battery of self-report questionnaires:

*Brief-COPE inventory* (Carver 1997; Conti 2000; Perna et al. 2007). It is a 28-item self-report measure of strategies used by individuals to cope with problems and stress, both adaptive and maladaptive. Each item can be answered on a four-point Likert-type scale ranging from “not at all” to “very much”. Finally the tool gives a score on 14 coping approaches namely:: acceptance, active coping, positive reframing, planning, use of instrumental support, use of emotional support, behavioral disengagement, self-distraction, self-blame, humor, denial, turning to religion, venting, and substance use.
*Symptom Check List 90-Revised (SCL-90-R)* (Derogatis et al. 1976; Conti 2000). The SCL-90-R is a 90-item scale used to evaluate a broad range of psychological problems and symptoms of psychopathology. It measures nine primary symptom dimensions (SOM: Somatization; O-C: Obsessive-Compulsive; I-S: Interpersonal Sensitivity; DEP: Depression ANX: Anxiety; HOS: Hostility; PHOB: Phobic Anxiety; PAR: Paranoid Ideation; PSY: Psychoticism) and three specific indexes assessing the global severity of symptoms [Global Severity Index (GSI) is designed to measure overall psychological distress, the Positive Symptom Distress Index (PSDI) measures the intensity of symptoms, and the Positive Symptom Total (PST) reports the number of self-reported symptoms]. The scale is designed to provide an overview of a patient’s symptoms and their intensity at a specific point in time, referred to the last week.
*Penn State Worry Questionnaire (PSWQ)* (Meyer et al. 1990; Fresco et al. 2003). It is a 16-item self-completion questionnaire which may be used as a screening device, for individuals with worry-related problems. Each item is rated on a scale from 1 (‘not at all typical of me’) to 5 (‘very typical of me’). Eleven items are worded in the direction of pathological worry, while the remaining five items are worded to indicate that worry is not a problem. Higher PSWQ scores reflecting greater levels of pathological worry; possible scores range from 16 to 80.
*Zung Anxiety Status Inventory (ASI)* (Zung 1971). It was developed by Zung as a clinician-administered rating instrument for anxiety symptoms. Twenty affective and somatic symptoms associated with anxiety are graded from 0 to 4 by an observer based on patient interview: the higher the score the greater the symptoms associated with anxiety. The ASI index converts the raw score by dividing the raw score by 80 then multiplying by 100. Raters (SS and PG) were specifically trained and showed a good inter-rate reliability on the instrument (*k* > 0.80).
*Zung Self-Rating Anxiety Scale (SAS)* (Zung 1971). It is a method of measuring levels of anxiety in patients who have anxiety-related symptoms. The SAS test is self-administered, with each response using a 4-point scale, from “none of the time” to “most of the time”. There are 20 questions with 15 increasing anxiety level questions and 5 decreasing anxiety questions.

### Statistical analyses

All data were entered in SPSS database (Version 13.0, SPSS Inc, Chicago, IL). An alpha = 0.05 was chosen for all statistical analyses.

Descriptive analyses were first performed on the sample. Nonparametric statistics were carried out, namely Chi-square test for categorical variables and Mann–Whitney test for continuous variables; correlations were calculated by means of Spearman’s coefficient. Linear regression was used for estimate of associations for continuous variables.

Factors were searched among the coping strategies by means of factorial analysis. We used a total sample size of 148 patients, with a ratio of items/cases = 1/10.57 (Gorsuch 1983; Tabachnick and Fidell 1989; Floyd and Widaman 1995). A principal components analysis with varimax rotation was used and factors were extracted by using Kaiser rule and Scree Test (Cattell 1978; Kline 1994; Matsunaga 2010). Brief-COPE items were included as part of the new coping scales if they met two basic criteria: (1) they loaded >0.30 on one of the factors, and (2) their loading on the factor was positive; when an item loaded >0.30 on more than one factors we assigned it to the factor with the highest loading value. Cronbach’s alpha for the new scales was calculated.

For each patient, the total scores for the factors were calculated by summing the scores on the constituent items. The so-obtained factor scores were used to analyze differences in coping strategies according to sociodemographic and clinical characteristics.

Finally, adjusted ORs with their 95% CI were calculated by means of binary logistic regression in a multivariate model.

## Results

### Sample characteristics

Two hundred two patients have been considered for the study; only 148 with an ADs were examined because they fulfilled the inclusion/exclusion criteria. Patients were on average 40 years old, in most cases they were females, Italians, married, or cohabitant, educated at high school level and employed. The mean age at the onset of AD was 30.53. The more frequent DSM-IV-TR anxiety disorder diagnosis was Generalized Anxiety Disorder (GAD) followed by Panic Disorder (PD), Anxiety Disorder Not Otherwise Specified (NOS), Adjustment Disorder (AD), Social Anxiety Disorder (SAD), and Obsessive-Compulsive Disorder (OCD). Thirty-four of the total sample underwent some previous psychotherapy.

Previous pharmacotherapy has been reported in 93 out of 148 patients (62.8%), with the following distribution among drug types: benzodiazepines (60.2%), SSRIs (22.7%), SNRIs (4.3%), tricyclic antidepressants (8.6%), antipsychotics (0.7%), mood stabilizers (2.2%), other (1.1%).

As for dimensional instruments, the mean score on the PSWQ was 46.3 ± 11.58, on the ASI was 52.79 ± 12.55, on the SAS was 57.59 ± 13.01. Finally, SCL-90-R higher scores were reported for the following dimension: Anxiety, Depression, Somatization, and Obsessive-Compulsive.

Sociodemographic, clinical, and lifestyle characteristics are reported in Table 2010, while scores to rating scales (PSWQ, ASI, SAS, and SCL-90-R) are shown in Table 2000.

**Table 1:** Sociodemographic and lifestyle characteristics of the sample (*N* = 148)

Age, M ± SD	40.77 ± 11.77
Gender	
Male	58 (39.2%)
Female	90 (60.8%)
Nationality	
Italian	141 (97.2%)
Other	4 (2.8%)
Years of education, M ± SD	13.45 ± 3.58
Occupational status	
Paid work	100 (67.6%)
Not paid work	5 (3.4%)
Housewife	16 (10.8%)
Retired from work	11 (7.4%)
Student	8 (5.4%)
Not employed	7 (4.7%)
Other	1 (.7%)
Marital status	
Not married	41 (28.3%)
Married	70 (48.3%)
Cohabitant	14 (9.7%)
Separated	11 (7.6%)
Divorced	5 (3.4%)
Widower	4 (2.8%)
Housing arrangements	
Alone	16 (11.2%)
Family of origin	22 (15.4%)
Proper family	98 (68.5%)
Other	7 (4.9%)
Age at the onset of disease, M ± SD	30.53 ± 11.72

M, Mean; SD, Standard deviation.

**Table 2:** Symptom scores achieved by patients (*N* = 148)

Rating scale	M ± SD	Ranges
PSWQ	46.3 ± 11.58	16–80
ASI	52.79 ± 12.55	29–90
SAS	57.59 ± 13.01	31–90
SCL-90-R		
Som	1.53 ± 0.87	0–4.7
O-C	1.48 ± 0.76	0.1–4.3
I-S	1.1 ± 0.82	0–4.3
Dep	1.69 ± 0.94	0.15–5.46
Anx	1.72 ± 0.88	0.3–5.2
Hos	1.04 ± 0.85	0–3.7
Phob	1.24 ± 1.1	0.5–1.57
Par	1.19 ± 0.87	0–4
Psy	0.82 ± 0.65	0–3.8
GSI	1.37 ± 0.69	0.3–4.6

PSWQ, Penn State Worry Questionnaire; ASI, Anxiety Status Inventory; SAS, Self-Rating Anxiety Scale; SCL-90-R, Symptom Check List 90-Revised; Som, Somatization; O-C, Obsessive-Compulsive; I-S, Interpersonal Sensitivity; Dep, Depression; Anx, Anxiety; Hos, Hostility; Phob, Phobic Anxiety; Par, Paranoid Ideation; Psy, Psychoticism; GSI, Global Severity Index; M, Mean; SD, Standard deviation.

### Factor analysis

Factor analysis yielded nine factors with eigenvalues greater than 1.0, which together accounted for 65.48% of the variance in responding. Factors loadings of Brief-COPE items are displayed in Table 2013. Factor 1 (Searching Support) included the following items: Get emotional support from others, Help and advice from others, Get comfort and understanding from someone, Advice/help from others about what to do, Say things to let feelings escape. Factor 2 (Problem Solving) included the following items: Take action to make situation better, Come up with strategy about what to do, Think about what steps to take. Factor 3 (Avoidance) included the following items: Say to myself: “This isn’t real”, Refuse to believe what has happened, Give up trying to deal with it, Give up attempt to cope. Factor 4 (Changing Perspective) included the following items: Look for something good in situation, Make jokes about situation, See in a different light to make seem more positive, Do something to think about it less. Factor 5 (Religion)included the following items: Find comfort in religious beliefs, Pray or meditate. Factor 6 (Acceptance) included the following items: Learn to live with situation, Accept reality that it has happened, Make fun of situation, Express negative feeling. Factor 7 (Substance use) included the following items: Use alcohol/drugs to feel better, Use alcohol/drugs to get through. Factor 8 (Not-finalized activity) included the following items: Turn to work or other activities to distract, Concentrate on doing something about situation. Factor 9 (Self-blame) included the following items: Criticize myself, Blame myself for things that happen.

**Table 3:** Factors loadings emerging from principal components analysis of Brief COPE items in anxiety patients (*N* = 148)

	Factor 1 Searching Support	Factor 2 Problem solving	Factor 3 Avoidance	Factor 4 Changing perspective	Factor 5 Religion	Factor 6 Acceptance	Factor 7 Substance use	Factor 8 Not-finalized activity	Factor 9 Self-blame
% total variance	17.06	9.78	7.62	6.55	6.03	5.21	4.68	4.43	4.10
Cronbach’s α	0.784	0.770	0.622	0.646	0.854	0.557	0.699	0.509	0.502
*Item*									
5. Get emotional support from others	0.815								
10. Help and advice from others	0.794								
15. Get comfort and understanding from someone	0.782								
23. Advice/help from others about what to do	0.660								
9. Say things to let feelings escape	0.471								
7. Take action to make situation better		0.821							
14. Come up with strategy about what to do		0.771							
25. Think about what steps to take		0.717							
3. Say to myself: “This isn’t real”			0.698						
8. Refuse to believe what has happened			0.665						
6. Give up trying to deal with it			0.636						
16. Give up attempt to cope			0.618						
17. Look for something good in situation				0.808					
18. Make jokes about situation				0.714					
12. See in a different light to make seem more positive				0.673					
19. Do something to think about it less				0.342					
22. Find comfort in religious beliefs					0.870				
27. Pray or meditate					0.863				
24. Learn to live with situation						0.684			
20. Accept reality that it has happened						0.678			
28. Make fun of situation						0.571			
21. Express negative feeling						0.515			
4. Use alcohol/drugs to feel better							0.878		
11. Use alcohol/drugs to get through							0.851		
1. Turn to work or other activities to distract								0.815	
2. Concentrate on doing something about situation								0.571	
13. Criticize myself									0.777
26. Blame myself for things that happen									0.604

Table 2013 also shows the value of Cronbach’s alpha for the items included in each of the 9 new coping scales across the total sample; their reliabilities all meet or exceed the value of 0.50 regarded as minimally acceptable (Nunnally 1978).

### Coping strategies according to calculated factors

Once the factors were extracted, the score of each factor was calculated in the whole sample. Patients scored higher on Searching Support (12.96 ± 3.83), followed by Acceptance (9.54 ± 2.65), Changing Perspective (9.32 ± 2.86), and Problem Solving (8.84 ± 2.39). The complete description of scores achieved by patients on each factor is reported in Table 2013.

**Table 4:** Coping strategies scores according to the nine factors extracted from the Brief-COPE in anxiety patients (*N* = 148)

Factor	M* ± *SD
Searching support	12.96 ± 3.83
Problem solving	8.84 ± 2.39
Avoidance	7.33 ± 2.78
Changing perspective	9.32 ± 2.86
Religion	4.75 ± 2.18
Acceptance	9.54 ± 2.66
Substance use	2.29 ± 0.98
Not-finalized activity	5.59 ± 1.75
Self-blame	5.6 ± 1.73

M, Mean; SD, Standard deviation.

### Differences in coping strategies according to sociodemographic characteristics

Coping styles differed significantly between females and males, with females using more Searching Support than males (13.57 ± 3.81 vs. 12.02 ± 3.7, *z* = −2.47, *P* = 0.014).

Years of education were associated positively with Changing Perspective (Spearman’s Rho = +0.176, *P* = 0.033; Beta = 0.14, 95% CI: 0.01–0.27). No significant differences in coping strategies were found with reference to age, nationality, occupational status, marital status, housing arrangements (data not shown).

### Differences in coping strategies according to clinical variables

No significant differences in coping strategies were found according to ADs diagnosis (categorized as follows: GAD, PD, Other ADs).

Age at onset of the disease was associated negatively with Searching Support (Spearman’s Rho = −0.244, *P* = 0.004; Beta = −0.077, 95% CI: −0.13; −0.02) and Self-blame (Spearman’s Rho = −0.194, *P* = 0.021; Beta = −0.026, 95% CI: −0.05; −0.002).

Coping styles differed significantly in patients who had previously undergone a psychotherapy when compared with patients who had not: the former used more Acceptance than the latter (10.12 ± 2.34 vs. 9.12 ± 2.61, *z* = −2.07, *P* = 0.04).

### Coping strategies and psychopathology

Searching support was associated positively with some psychopathology scores, namely Penn State Worry Questionnaire, Anxiety Status Inventory, Self-Rating Anxiety Scale, and the following SCL-90-R subscales: Obsessive-Compulsive, Interpersonal Sensitivity, Depression, Anxiety, Phobic Anxiety, Paranoid Ideation. Furthermore, Avoidance coping, was associated positively with psychopathology scores, namely scores on Penn State Worry Questionnaire, Anxiety Status Inventory, Self-Rating Anxiety Scale, and the following SCL-90-R subscales: Somatization, Obsessive-Compulsive, Interpersonal Sensitivity, Depression, Anxiety, Hostility, Phobic anxiety, Paranoid Ideation, Psychoticism. A positive association between Religion and Penn State Worry Questionnaire score was found too.

Regarding the associations with SCL-90-R, Self-blame was positively associated with both Interpersonal sensitivity and Paranoid Ideation. Problem Solving was negatively associated with Anxiety Status Inventory and Self-Rating Anxiety Scale score. Finally, Changing Perspective was negatively associated with Self-Rating Anxiety Scale score. Values of Spearman’s Rho and Beta coefficients with 95% CI for the above mentioned associations are not shown.

When dichotomizing patients according to GSI score (<1 vs. ≥1), Avoidance was significantly higher in patients with GSI ≥ 1 (8.12 ± 2.87 vs. 5.82 ± 1.64, *z* = −4.730, *P* < 0.001), whilst Problem Solving was higher in patients with GSI < 1 (9.56 ± 2.08 vs. 8.46 ± 2.43, *z* = −2.61, *P* = 0.009).

### Prediction of psychopathology by means of multivariate analysis

When taking into account predictors of psychopathology in a multivariate model, significant predictors of the GSI score resulted: gender, PSWQ score, and Problem-solving coping strategy; namely, the female gender and a higher PSWQ score increased the risk of having a GSI score ≥1, with an adjusted OR of 2.444 (95% CI: 1.072–5.573) and 1.071 (95% CI: 1.030–1.115) respectively, whilst the use of a problem-solving coping strategy reduced the risk of having a GSI score ≥1 (adjusted OR = 0.780, 95% CI: 0.646–0.942).

## Discussion

To the best of our knowledge, this is the first study performing a factorial analysis of coping styles using the Brief-COPE in a population of Italian patients with ADs.

Nine coping styles are reported in our sample of anxiety outpatients, occurring in the following order of frequency: searching support, acceptance, changing perspective, problem solving, avoidance, self-blame, not-finalized activity, religion, substance use. Overall, our results are in partial agreement with previous factor analytic findings, suggesting the presence of broader underlying dimensions of coping (e.g., Fillion et al. 2002; Zuckerman and Gagne 2003; Kapsou et al. 2010). Indeed, our results show that some factors consisted of two items (tracing Carver’s Brief-COPE factors), while some others contained clusters of several items; thus, religion, substance use, and self-blame scales emerged as independent factors, which is in partial agreement with Miyazaki et al. (2008), Fillion et al. (2002), and Kapsou et al. (2010). Similarly to what previously reported by Kapsou et al. (2010), emotional and instrumental support coping styles loaded together on a factor (searching support). Strategies including planning, positive reframing and acceptance did not cluster together, as reported by Miyazaki et al. (2008) and Kapsou et al. (2010) (“active/positive coping”) but loaded on three different factors (problem solving, changing perspective, and acceptance). The avoidance factor included denial and behavioral disengagement. As to our results, venting and humor didn’t load together on a single factor. Thus, the data of the present study support a nine-factor structure of the Brief-COPE that includes five broad dimensions of coping.

According to the correlations performed in our sample, psychopathology as assessed by SCL-90-R, SAS, ASI, and PSWQ, is related mostly to Searching support and Avoidance factors, showing that these strategies may reflect ineffective ways of coping. On the other side, Problem solving and Changing perspective could be a valid approach to moderate anxiety symptoms and psychopathology in general. This is also supported by the multivariate model showing that a coping strategy based on problem solving is protective for psychopathology, after adjusting for gender and worrying (as assessed by PSWQ scores). Avoidant and emotion-focused coping has been previously linked to anxiety (Davey et al. 1995; Hayes et al. 1996; Feldner et al. 2004), whilst active and problem-focused strategies have been associated with better health outcomes (Penley et al. 2002). Findings in the literature have generally shown that emotion-focused coping is predictive of higher levels of psychopathology and functional impairment (e.g., Kohn et al. 1994; Ravindran et al. 1996). In particular, emotion-focused coping strategies such as avoidance, self-blame, venting, and rumination are associated with higher levels of anxiety, depression and distress in both nonclinical (Whatley et al. 1998; Aldao et al. 2010) and clinical samples (Ravindran et al. 1996; Aldao et al. 2010).

In our sample, women used Searching support significantly more frequently than men, as other studies reported (e.g., Kelly et al. 2007); indeed, there is similar evidence showing that women may be more likely than men to employ emotion-focused and avoidance coping (e.g., Kelly et al. 2007; Eaton and Bradley 2008; Kapsou et al. 2010), which may be partly responsible for their higher propensity toward depression, anxiety and other emotional disorders.

In addition, patients who underwent psychotherapy used more Acceptance than those who did not, suggesting the suitability of psychosocial interventions to develop coping strategies (Taylor and Stanton 2007; Wesner et al. 2014a).

Kramer et al. (2013) reported reduction in unhelpful coping strategies after Brief Psychodynamic intervention. Wesner et al. (2014b) evaluated the effect of cognitive-behavioral therapy on the choice of coping strategies in Panic Disorder patients, reporting an increased use of a more adaptive coping after psychotherapy.

Taking into account broader factors in coping strategies could help addressing extended modalities of dysfunctional coping when patients receive psychotherapeutic treatments and/or selecting which patients would benefit from a specific psychotherapeutic technique.

Some limitations of this study must be considered. The use of self-administered questionnaires to identify coping strategies and to assess psychopathology may raise an issue of comprehension and self-recognition. Although the exclusion of comorbidity in our sample maybe regarded as a point of strength, it is also difficult to ascertain what is the direction of causality (i.e., psychopathology leading to poor coping, or *vice versa*) due to our cross-sectional study design. Furthermore, a possible variability in coping strategies may be addressed to different anxiety sub-groups, considering the fact that many ADs have not so defined borders.

All these points should be addressed in further studies, since in-depth knowledge about coping styles among anxiety patients may provide better understanding of mechanisms underlying the pathogenesis of ADs, and clues for the advancement of prevention, treatment, and rehabilitation approaches as well.

## Conflict of Interest

All Authors declare no conflict of interest or financial support.
